# Morbidity and mortality after esophagectomy for esophageal carcinoma: A risk analysis

**DOI:** 10.1186/1477-7819-3-37

**Published:** 2005-06-21

**Authors:** Ines Gockel, Christoph Exner, Theodor Junginger

**Affiliations:** 1 Department of General and Abdominal Surgery, Johannes Gutenberg University of Mainz, Germany

## Abstract

**Background:**

The study was aimed to identify pre- and intraoperative risk factors that potentially influence morbidity and mortality after esophagectomy for esophageal carcinoma with particular emphasis on the predominant tumor types.

**Patients and methods:**

Between September 1985 and March 2004, 424 patients underwent esophagectomy for esophageal carcinoma. Of these, 186 (43.9%) patients had a transhiatal, and 231 (54.5%) patients underwent a transthoracic procedure with two-field lymphadenectomy. Pre-, intraoperative risk factors and tumor characteristics were included in the risk analysis to assess their influence on postoperative morbidity and mortality.

**Results:**

Multivariate analysis (logistic regression model) identified the surgical procedure as the most important risk factor for postoperative morbidity and mortality with the transthoracic technique associated with a significant higher risk. The comparison of the risk profile between the different histological tumor types, a significantly higher nutritional risk, poorer preoperative lung function and a higher prevalence of hepatopathy was observed in patients with squamous cell carcinoma (n = 229) compared to adenocarcinoma (n = 150) (p < 0.05). Although there was no significant difference in surgical complications between the two groups, the rate of general complications, length of postoperative intensive care unit-stay and mortality rate was significantly higher in patients with squamous cell carcinoma (p < 0.05).

**Conclusion:**

The present risk analysis shows that the selection and the type of the surgical procedure are crucial factors for both the incidence of postoperative complications and the mortality rate. The higher risk of the transthoracic procedure is justified with a view to a better long term prognosis.

## Background

Despite the standardization of the operative technique, improvement of preoperative risk assessment, and postoperative intensive care management, surgical therapy for esophageal carcinoma continues to be associated with a high incidence of operative complications, and a high mortality rate. Risk stratification in the selection of patients for surgery, and the choice of the surgical procedure are therefore important considerations.

The present study was aimed to identify preoperative and intraoperative factors that could potentially influence morbidity and mortality after esophagectomy for esophageal carcinoma.

## Patients and methods

Between September 1985 and March 2004, a total of 424 patients underwent esophagectomy for esophageal carcinoma in the Department of General and Abdominal Surgery of the Johannes Gutenberg-University Hospital Mainz. Of these, 186 (43.9%) patients had transhiatal esophagectomy, 231 (54.5%) patients underwent abdominothoracic esophagectomy with two-field lymphadenectomy (abdominal and mediastinal) and 7 patients had a proximal esophageal resection with a free jejunal graft. Reconstruction was accomplished by gastric tube pull-up in 384 (93.0%) patients, by colon interposition in 21 (5.1%), and small intestine interposition in 7 (1.7%) patients; the anatomic prevertebral esophageal bed was used for the majority of these procedures. Extra-anatomic reconstruction by the retrosternal route with cervical anastomosis after pull-up was carried out in patients with a high risk for loco-regional recurrence only (n = 42; 10.2%).

Abdominothoracic esophagectomy was routinely performed for squamous cell carcinoma (n = 229). A transhiatal procedure was selected for tumors with a distal location, for malignancies without esophageal wall penetration, or in the presence of a high general risk. Transhiatal esophagectomy with abdominal and posterior mediastinal lymphadenectomy was carried out for the majority of adenocarcinomas (n = 150), the two-field procedure was performed in the presence of advanced tumor growth or extended lymph node involvement. Long-term results of this choice of the operative procedure adjusted to the histological tumor type had shown a significant prognostic advantage in patients undergoing transthoracic compared to transhiatal resection in squamous cell carcinoma whereas there was no survival benefit in patients with adenocarcinoma of the esophagus [Junginger T, et al.; Gockel I et al.; unpublished data].

Data were collected prospectively in a specially established database. Preoperative and intraoperative variables as well as postoperative morbidity and mortality were documented, in addition to routine demographic data.

The following **preoperative risk factors **were recorded: ASA-classification (I-IV) according to the preoperative anesthesiology evaluation, BMI (Body Mass Index) based on body weight and height in kg/m^2^, and the nutritional status on a scale from 0 (= no alcohol or tobacco consumption), 1 (= tobacco alone), 2 (alcohol alone) to 3 (= combined nutritional risk with tobacco and alcohol use). Among the preoperative diseases, cardiovascular risk factors were defined as a history of coronary heart disease, or myocardial infarction, arterial hypertension, valvular disease (>II°), arrhythmia requiring therapy (>III° according to the Lown-classification), congestive heart failure NYHA (New York Heart Association) > Grade II, and peripheral occlusive arterial disease (>IIb according to Fontaine). A history of chronic obstructive pulmonary disease (COPD), regular tobacco consumption and/or the use of bronchodilators were subsumed under pulmonary diseases. The preoperative assessment of the vital capacity (VC) and forced expiratory volume (FEV_1 _= Tiffeneau-test) served to ensure a more accurate assessment. Prior cirrhosis of the liver (>/= CHILD-Pugh A) was defined as hepatic disease, and determined on the basis of the assessment of serum albumin (g/dl), serum bilirubin (mg/dl), Quick-value (%), and the presence of ascites or encephalopathy. The evaluation of additional risk factors included the prevalence of diabetes mellitus (insulin-dependent or requiring oral antidiabetic therapy), and the history of a secondary carcinoma.

Intraoperative variables included in the risk analysis were tumor location, the operative procedure, and the transfusion requirement of packed erythrocytes. The group of tumor characteristics comprised tumor size, R-classification, TNM-stage, and the number of dissected lymph nodes.

Postoperative variables were not part of this risk analysis. Morbidity, surgical (anastomotic leakage, graft necrosis, mediastinitis, recurrent laryngeal nerve paralysis, chylothorax, tracheal fistula, bleeding requiring reoperation) and general complications (pneumonia, atelectasis, ARDS i.e. adult respiratory distress syndrome, myocardial infarction, cardiac failure, pulmonary embolism, renal insufficiency, pancreatitis, deep vein thrombosis), length of intensive care unit stay, 30-day mortality, and the mortality rate served as factors for assessment.

### Statistical analysis

The SSPS 10.0 software package was used for statistical data analysis (SSPS, Chicago, IL, USA: 1999). Data are expressed as median with ranges (minimum – maximum), or as percentages (%).

Factors with a possible influence on perioperative morbidity and mortality were calculated using the logistic regression model (univariate and multivariate). The χ^2 ^test with Pearson's correction and Fisher's exact test were used for comparison of the parameters for squamous cell carcinoma and adenocarcinoma. The Mann-Whitney U test served as the non-parametric method. A *p *-value of <0.05 was considered statistically significant for all procedures.

## Results

### Preoperative and intraoperative parameters, tumor characteristics

Median patient age at the time of surgery was 58 (28–84) years; the proportion of males was 83.0%. The median BMI (Body Mass Index) was 24.4 (13.8–39.3) kg/m^2^. A total of 36.9% patients were classified as ASA grade II, 58.2% as ASA grade III, and 4.9% as ASA grade IV. A combined nutritional risk (alcohol and tobacco use) was determined in 41.5% of patients. Tobacco use alone was found in 59.1%, and habitual alcohol consumption in 58.2% of patients. Preexisting cardiovascular diseases were noted in 28.2%, pulmonary disease in 13.5%, hepatopathy in 4.9%, and diabetes mellitus in 5.9% of patients undergoing surgery for esophageal carcinoma. The history of 9.8% of all patients showed the presence of secondary carcinoma. Median preoperative vital capacity (VC) ranged at 3.8 (1–7.2) l, FEV_1 _(Tiffeneau-test) was 2.9 (0.5–9.0) l/sec.

Out of all tumors, 56.3% were located in the lower, 34.6% in the middle, and 9.1% in the upper third of the esophagus. Squamous cell carcinoma was identified in 55.3%, adenocarcinoma in 35.9%, and an undifferentiated carcinoma in 7.6% of patients (1.2% with other malignant esophageal tumors, e.g. melanoma) who underwent esophagectomy.

Operative time ranged at 300 (160–560) minutes. The median number of packed erythrocyte units used was 1 (0–38) (54.6% of all patients did not require packed erythrocyte transfusion). Median length of intensive care therapy was 10 (0–176) days, at a total postoperative hospital stay of 22 (0–189) days. Median tumor size was 4 (0.3–20) cm. A R0 resection was accomplished in 81.0% of all patients (R1 resection: 16.0%, R2 resection: 3.0%). Based on results of the pathological examination, the majority of tumors were assigned to the T3 category (57.5%), while 34.6% of all tumors were allocated to the T1 or T2, and 7.9% to the T4-category. Distribution for the N category was as follows: N0: 34.0%, N1: 66.0%. A M1-situation (M1-lymph node or M1-organ) was identified in 26.5% of all patients, which was treated with curative intent in the course of the same surgical procedure.

### Morbidity and mortality: Prevalence and influence factors

There was a 35.5% prevalence of surgical complications in the total population of 424 patients undergoing esophagectomy for esophageal carcinoma. General complications were observed in 36.0% of all patients. The 30-day mortality rate was 6.7%, at a mortality rate for the entire observation period of 11.5%.

Anastomotic leakage was the most common surgical complication (18.2%), followed by recurrent laryngeal nerve paresis (15.7%). There was a similar incidence of graft necrosis (3.2%) and postoperative hemorrhage (2.9%) requiring surgical revision, while 1.3% of all patients developed a chylothorax.

Univariate analysis showed tumor location (p = 0.0194) and the surgical procedure (p = 0.0116) to be significantly related to the incidence of surgical complications; none of the other pre- and intraoperative factors, including tumor characteristics, were of relevance in this model (Table 1). Multivariate analysis identified the transthoracic surgical procedure (p = 0.0004), tumor location (upper third) (p = 0.02), and the transfusion of packed erythrocytes (p = 0.04) as factors significantly related to the incidence of surgical complications (Table 2).

**Table 1:** Factors with a potential influence on surgical and general complications, as well as on the perioperative mortality rate after esophagectomy for esophageal carcinoma: univariate analysis (p values).

	**Surgical complications**	**General Complications**	**Perioperative Mortality**
Preoperative factors:			
- Age	0.8996	0.0073*	0.1349
- Sex	0.7367	0.3396	0.5658
- BMI	0.3710	0.1200	0.0719
- ASA	0.3992	0.0076*	0.0274*
- Nutritional status	0.5093	0.1897	0.2223
- Cardiovascular PD	0.7803	0.0171*	0.0172*
- Pulmonary PD	0.2910	0.0405*	0.0059*
- Hepatopathy	0.1696	0.0282*	0.0165*
- Diabetes mellitus	0.8218	0.0635	0.9430

Intraoperative factors:			
- Tumor location	0.0194*	0.0156*	0.0391*
- Surgical procedure	0.0116*	0.0001*	0.0057*
- Packed erythrocyte transfusion	0.1045	0.0006*	0.0022*

Tumor characteristics:			
- Tumor size	0.5041	0.1595	0.1502
- R-classification	0.5573	0.3372	0.0439*
- T-category	0.2017	0.0072*	0.0036*
- N-category	0.6805	0.9928	0.0584
- M-category	0.0621	0.8128	0.4359

*statistically significant

**Table 2:** Significant factors with a potential influence on surgical and general complications, as well as on the perioperative mortality rate after esophagectomy for esophageal carcinoma: multivariate analysis (p value).

**Surgical complications**	**General complications**	**Perioperative Mortality**
Surgical procedure (p = 0.0004)	Surgical procedure (p = 0.0001)	Surgical procedure (p = 0.0068)
Tumor location (p = 0.0204)	Packed erythrocyte transfusion (p = 0.0009)	Pulmonary PD (p = 0.0096)
Packed erythrocyte transfusion (p = 0.0493)	Patient age (p = 0.0039)	Packed erythrocyte transfusion (p = 0.0099)
	Nutritional status (p = 0.0284)	
	ASA-classification (p = 0.0486)	

Pulmonary complications were most common among the general complications (32.9%). Adult respiratory distress syndrome (ARDS) occurred in 6.5% of patients. Postoperatively, 3.7% of patients developed renal failure requiring dialysis, and myocardial infarction or pulmonary embolism occurred in 1.2%, respectively, of all patients. Postoperative pancreatitis was found in 0.5% of patients. Univariate analysis determined patient age (p = 0.0073), ASA-classification (p = 0.0076), preexisting cardiovascular (p = 0.0171), pulmonary (p = 0.0405), and hepatic (p = 0.0282) disease as significant preoperative variables in relation to postoperative general complications. Similarly significant among the intraoperative factors were tumor location (p = 0.0156), the surgical procedure (p = 0.0001), transfusion of packed erythrocytes, as well as the T-category in the group of tumor characteristics (Table 1). On multivariate analysis, independent parameters with a significant influence on postoperative general complications were in decreasing order: the surgical procedure (p = 0.0001), transfusion of packed erythrocytes (p = 0.0009), patient age (p = 0.0039), nutritional status (p = 0.0284), and ASA-classification (p = 0.0486) (Table 2).

The cause of postoperative mortality (11.5%; n = 49 patients) in 19 patients was sepsis (accounted for by anastomotic insufficiency in 8, and in 3 patients by graft necrosis). Twelve patients died from pulmonary failure, and 5 patients died after myocardial infarction. Fulminant pulmonary embolism was the cause of death in 3 patients (one out of these occurred in a patient with suture dehiscence). Two patients developed hemorrhagic shock, and another 2 patients died in hospital from rapid progression of the malignant disease. Further causes of hospital death in 6 additional patients after esophagectomy were; hepatorenal syndrome, syncopal attack with cardiovascular failure, right ventricular heard failure, medial cerebral infarction, intraoperative cardiac arrest, and tracheogastric fistula.

Preoperative variables as predictors of a fatal postoperative course found on univariate analysis comprised ASA-classification (p = 0.0274), preexisting cardiovascular (p = 0.0172) and pulmonary disease (p = 0.0059), and hepatopathy (p = 0.0165). Further factors exerting an influence on mortality were: tumor location (p = 0.0391), the surgical procedure (p = 0.0057), packed erythrocyte transfusion (p = 0.0022), R-classification (p = 0.0439), and T-category (p = 0.0036) (Table 1). Multivariate analysis identified the surgical procedure (p = 0.0068), preexisting pulmonary disease (p = 0.0096), and the transfusion of packed erythrocytes (p = 0.0099) as the most significant predictors of mortality (Table 2).

### Risk profile: comparison between squamous cell carcinoma and adenocarcinoma

From among the group of preoperative factors, patients with adenocarcinoma were characterized by significantly more advanced age and a higher BMI (p < 0.0001) compared to those with squamous cell carcinoma. Differences in gender distribution and ASA-classification were not significant. There was a significantly higher nutritional risk (alcohol and tobacco use) in patients with squamous cell carcinoma than in the comparison group of patients with adenocarcinoma undergoing esophagectomy (p < 0.001). The incidence of cardiovascular and pulmonary disease, as well as preexisting diabetes mellitus, or secondary carcinoma in the patient history was similar in both groups. However, patients with squamous cell carcinoma had significantly poorer preoperative lung function (p = 0.0078), and a higher prevalence of hepatopathy (p < 0.0001) (Table 3).

**Table 3:** Comparison of the preoperative risk profile between patients with squamous cell carcinoma and adenocarcinoma of the esophagus.

**Parameter**	**Squamous cell carcinoma (n = 229)**	**Adenocarcinoma (n = 150)**	**p-value**
- Age (years)	56.0 (29–84)	61.0 (28–78)	0.0001*
- Sex (% males)	79.8	87.4	0.071
- BMI (kg/m^2^)	23.2 (13.9–34.9)	25.8 (15.8–39.3)	0.0001*
- ASA-classification (%)			
-II	33.2	43.2	
-III	62.4	52.1	0.072
-IV	4.4	4.8	
- Nutritional risk (%)			
-No nutritional risk (%)	16.2	43.2	0.0001*
-Alcohol use (%)	69.5	42.9	0.0001*
-Tobacco use (%)	73.5	38.1	0.0001*
-Combined risk (%)	83.8	56.9	0.0001*
- Cardiovascular PD (%)	30.4	24.7	0.240
- Pulmonary PD (%)	14.7	14.4	0.593
-VC (l)	3.7 (1.0–6.6)	3.8 (1.9–6.1)	0.3063
-FEV 1 (l/sec)	2.8 (0.5–5.2)	3.1 (1.3–5.9)	0.0078*
- Hepatopathy (%)	8.1	1.4	0.0001*
- Diabetes mellitus (%)	4.9	7.5	0.369
- Secondary carcinoma (%)	11.2	8.2	0.382

*statistically significant

The surgical procedure used was different with respect to the significantly more frequent performance of transhiatal esophagectomy in patients with adenocarcinoma (p < 0.0001). In the presence of this histological subtype, the gastric tube (p = 0.027) brought up in the existing esophageal bed (p = 0.001) was used more often as the interposed organ. The number of dissected abdominal lymph nodes was significantly higher (p = 0.0146) for adenocarcinoma, while a significantly higher number of thoracic lymph nodes (p = 0.0011) was removed in patients with squamous cell carcinoma. The operative time was significantly shorter (p < 0.0001) at a lower packed erythrocyte transfusion requirement for adenocarcinoma (p = 0.0495) compared to squamous cell carcinoma (Table 4). There was no significant difference between the two groups with regard to UICC stages and tumor characteristics (p > 0.05).

**Table 4:** Intraoperative Factors: Comparison between squamous cell carcinoma and adenocarcinoma of the esophagus.

**parameter**	**Squamous cell carcinoma (n = 229)**	**Adenocarcinoma (n = 150)**	**p-value**
- Surgical procedure (% transhiatal)	30.6	68.7	0.0001*
- Esophageal substitute (% gastric tube)	90.8	97.3	0.027*
- Repositioning (% esophageal bed)	83.7	95.3	0.001*
- Number of removed abdominal LN (n)	11 (0–55)	13 (0–51)	0.0146*
- Number of removed thoracic LN (n)	11 (0–47)	6 (0–83)	0.0011*
- Operative time (min)	305 (115–560)	270 (160–540)	0.0001*
- Intraoperative blood loss (ml)	1000 (200–5000)	800 (0–7500)	0.2224
- Units of transfused packed erythrocytes (n)	1.5 (0–38)	0 (0–14)	0.0495*

*statistically significant

Although there was no significant difference in surgical complications between the two groups, the rate of general complications (p = 0.012), and thus the total complication rate, (p = 0.039) was significantly higher in patients with squamous cell carcinoma. Comparable to the morbidity rate, the duration of postoperative intensive care therapy was significantly longer (p < 0.0001) for this type of tumor than for adenocarcinoma.

The 30-day mortality rate was identical for both groups, while the mortality rate in patients with squamous cell carcinoma was significantly higher (p < 0.0001) (Table 5).

**Table 5:** Postoperative morbidity and mortality for squamous cell carcinoma compared with adenocarcinoma of the esophagus.

**parameter**	**Squamous cell carcinoma (n = 229)**	**Adenocarcinoma (n = 150)**	**p-value**
- Surgical complications (%)			
- General complications (%)	37.9	33.7	0.447
-Total complications (%)	40.7	28.0	0.012*
- Length of ICU – stay (d)	68.1	57.6	0.039*
- 30-day-moratlity (%)			
- Mortality (%)	11 (2–176)	8 (1–107)	0.0001*
	6.1	6.1	0.8569
	15.1	6.6	0.0001*

*statistically significant

## Discussion

Although the morbidity rate after esophagectomy for esophageal carcinoma has been markedly reduced in recent years as a result of improvements in patient selection, surgical technique, and advances in perioperative management, the morbidity rate remains high [1-7]. Pulmonary function disturbances are the major contributing factor here [8-10]. Risk factors for the development of impaired pulmonary function include smoking, patient age (>70 years), obesity, and preexisting COPD [11]. Parameters with an influence on perioperative mortality defined by Bartels *et al *, are in decreasing order: reduced general status of the patient, impaired cardiac and hepatic function, and respiratory function [12]. The evaluation of the preoperative function of the described organ systems using a scoring system developed by the authors enables the classification of risk groups, and leads to a decrease in the operative risk as a result of adequate patient selection [12].

The aim of our analysis of data collected prospectively in 424 patients undergoing surgery for esophageal carcinoma was to answer the question as to what extent the selection of the surgical procedure, i.e. the transthoracic or the transhiatal approach, and the operative course assessed on the basis of intraoperative blood loss, in addition to patient and tumor related factors, exert an influence on the postoperative course.

The data were collected in 424 consecutive patients undergoing surgery for esophageal carcinoma over the period from 1985 to 2004. Surgical complications occurred in 35.5% and general complications in 36.0% of patients. The 30-day mortality rate ranged at 6.7%, at a mortality rate of 11.5%. Univariate analysis identified the selection of the surgical procedure as the main risk factor affecting the mortality rate. Transthoracic esophagectomy was associated with a higher complication and mortality rate than transhiatal dissection. Various authors have investigated the question as to the effectiveness of an extended radical procedure with an associated increased operative risk after the transthoracic technique. Results of two randomized studies did not demonstrate a difference between these procedures, although the meaningfulness of these findings is limited due to the small number of patients enrolled and the lack of information on the oncological radicality, especially the extent of lymph node dissection [13,14]. In addition, only patients with early tumor stages [14] or with a distal location of the carcinoma [13] were taken into consideration, respectively.

A higher pulmonary complication rate after the transthoracic compared to the transhiatal procedure for adenocarcinoma was found by a prospective randomized study [15]. Postoperative ventilation time, intensive care unit and postoperative in-hospital stay, reflecting perioperative morbidity, were significantly longer after transthoracic in contrast to transhiatal esophagectomy in this trial. However, the authors did not determine any differences in the long-term course [15]. This prospective study confirmed the outcome of a meta analysis revealing significantly higher early (pulmonary) morbidity and mortality after the transthoracic procedure published previously by the same author with 5-year survival rates of approximately 20% after both kinds of resection [16]. In our patient population, the long-term prognosis for patients with squamous cell carcinoma undergoing transthoracic surgery – though exhibiting a higher perioperative morbidity and mortality – was significantly better than that for patients after transhiatal resection [Junginger T, et al.; unpublished data]. Patients with adenocarcinoma did not differ in survival undergoing transhiatal or transthoracic esophagectomy [Gockel I, et al; unpublished data]. In concert with other authors, we therefore favor the transhiatal technique with posterior mediastinal and upper abdominal lymph node dissection for adenocarcinoma of the esophagus, and the transthoracic procedure with abdominal and mediastinal lymphadenectomy for squamous cell carcinoma. Thus – long term results regarding the surgical technique of our own patient population have to be viewed critically, especially for adenocarcinoma, as the two groups differed significantly in UICC-stage and R-classification in contrast to patients with squamous cell carcinoma with a rather equal distribution [1,2].

Independent of the operative procedure, surgical blood loss had a significant influence on the postoperative morbidity and mortality rates. This is in accordance with experiences reported by Whooley *et al *[8], and indicates that the selection of a limited resection technique can be crucial for the development of the postoperative course. Patient-associated parameters (age, nutritional status, ASA-classification) were of relevance only with respect to the occurrence of general complications.

Preexisting pulmonary disease was an independent predictor of postoperative mortality. This confirms the findings of Chan *et al *, who identified impaired pulmonary function as a preoperative variable predictive of postoperative mortality [17]. The implementation of an appropriate preoperative therapy, discontinuation of smoking, more frequent use of epidural analgesia, and early bronchoscopy in the presence of the suspicion of postoperative pulmonary secretion impairment are essential factors for risk reduction [8].

In contrast to results of risk analyses by Law *et al *[18] and Lund *et al *[19], tumor characteristics as, e.g. TNM classification, were of no influence on the postoperative course in the patient population of this study.

A different operative risk was determined for the two histological tumor types of the esophagus: while there were similar surgical complications in both groups, overall morbidity and mortality rates were significantly higher in patients with squamous cell carcinoma than in the group with adenocarcinoma. In accordance with reports in the literature [20-22], this reflects, on the one hand, the different preoperative risk profile of both entities, consisting of an increased nutritional risk, higher prevalence of hepatopathy, and poorer lung function in patients with squamous cell carcinoma. Additionally, in this study there was a significantly higher incidence of transthoracic esophagectomy with a higher complication rate in patients with squamous cell carcinoma than in those with adenocarcinoma (69.4 vs. 31.3%).

## Conclusion

The present analysis shows that the selection and the type of the surgical procedure are crucial factors for both the incidence of postoperative complications and the mortality rate. The transhiatal procedure is associated with a significantly lower morbidity and mortality rate and thus represents – as long term survival does not favor the transthoracic approach – the surgical technique of choice for adenocarcinoma of the esophagus. In contrast, our previous long-term experience and results obtained by this study advocate the performance of the transthoracic procedure for squamous cell carcinoma. The higher operative risk is justified with a view to a better long-term prognosis. Independent of the choice of the operative approach, a less-invasive surgical procedure and the implementation of measures designed to minimize the risk of pulmonary complications are essential to achieve a reduction in the morbidity rate of esophageal carcinoma.

## Competing interests

The author(s) declare that they have no competing interests.

## Authors' contributions

**IG: **study design, collection of data, statistical analysis, sequence alignment, draft of manuscript

**ChE: **collection of data, statistical analysis

**ThJ: **conceived of the study, design and coordination of the study, draft and revision of the manuscript
